# Apolipoprotein E deficiency potentiates macrophage against *Staphylococcus aureus* in mice with osteomyelitis via regulating cholesterol metabolism

**DOI:** 10.3389/fcimb.2023.1187543

**Published:** 2023-07-17

**Authors:** Mincheng Lu, Ruiyi He, Chao Li, Zixian Liu, Yuhui Chen, Bingsheng Yang, Xianrong Zhang, Bin Yu

**Affiliations:** ^1^ Division of Orthopedics and Traumatology, Department of Orthopedics, Nanfang Hospital, Southern Medical University, Guangzhou, Guangdong, China; ^2^ Guangdong Provincial Key Laboratory of Bone and Cartilage Regenerative Medicine, Nanfang Hospital, Southern Medical University, Guangzhou, Guangdong, China

**Keywords:** Apolipoprotein E, Staphylococcus aureus, osteomyelitis, infection, cholesterol metabolism, macrophages

## Abstract

**Introduction:**

*Staphylococcus aureus* (*S. aureus*) osteomyelitis causes a variety of metabolism disorders in microenvironment and cells. Defining the changes in cholesterol metabolism and identifying key factors involved in cholesterol metabolism disorders during *S. aureus* osteomyelitis is crucial to understanding the mechanisms of *S. aureus* osteomyelitis and is important in designing host-directed therapeutic strategies.

**Methods:**

In this study, we conducted *in vitro* and *in vivo* experiments to define the effects of *S. aureus* osteomyelitis on cholesterol metabolism, as well as the role of Apolipoprotein E (ApoE) in regulating cholesterol metabolism by macrophages during *S. aureus* osteomyelitis.

**Results:**

The data from GSE166522 showed that cholesterol metabolism disorder was induced by *S. aureus* osteomyelitis. Loss of cholesterol from macrophage obtained from mice with *S. aureus* osteomyelitis was detected by liquid chromatography-tandem mass spectrometry(LC-MS/MS), which is consistent with Filipin III staining results. Changes in intracellular cholesterol content influenced bactericidal capacity of macrophage. Subsequently, it was proven by gene set enrichment analysis and qPCR, that ApoE played a key role in developing cholesterol metabolism disorder in *S. aureus* osteomyelitis. ApoE deficiency in macrophages resulted in increased resistance to *S. aureus*. ApoE-deficient mice manifested abated bone destruction and decreased bacteria load. Moreover, the combination of transcriptional analysis, qPCR, and killing assay showed that ApoE deficiency led to enhanced cholesterol biosynthesis in macrophage, ameliorating anti-infection ability.

**Conclusion:**

We identified a previously unrecognized role of ApoE in *S. aureus* osteomyelitis from the perspective of metabolic reprogramming. Hence, during treating *S. aureus* osteomyelitis, considering cholesterol metabolism as a potential therapeutic target presents a new research direction.

## 1 Introduction

Osteomyelitis, an infection of bone tissue and bone marrow caused primarily by microbial pathogens, is becoming an increasingly serious health problem. Since the 1970s, there have been less improvement in surgical techniques to reduce the incidence of osteomyelitis, resulting in a continued slow increase in the rate of infection in the hip and knee after open fracture surgery, reaching 5-33%, and 1-4% after arthroplasty (Acharya et al., 2013; Metsemakers et al., 2018; Schwarz et al., 2019). *S. aureus* and coagulase-negative staphylococci account for two-thirds of all osteomyelitis pathogens, with *S. aureus* being the most common single pathogen (Masters et al., 2022). The bone destruction observed during osteomyelitis suggests that *S. aureus* utilizes a dynamic nutrient environment inside the host, as host consumption and release of nutrients are altered by widespread cell death and inflammation (Potter et al., 2020).

Macrophages rely on pattern recognition receptors (PRRs) and other similar receptors to recognize pathogen-associated molecular patterns (PAMPs) (Ozinsky et al., 2000; Takeda et al., 2003). Rapid recognition of foreign factors leads to the production of pro-inflammatory cytokines and chemokines, phagocytosis, reactive oxygen species (ROS), and recruitment of other immune cells to the site of infection (Wynn et al., 2013). Macrophages can rapidly reprogram their metabolic state to promote inflammation and effector function (Hubler and Kennedy, 2016; Russell et al., 2019). However, *S. aureus* can lead to a metabolic reprogramming disorder in macrophages. Fumarate, which is itself a glycolytic inhibitor (Soh et al., 2020), induces epigenetic changes in macrophages that promote trained immunity, enhancing cytokine production (Berends et al., 2019). Furthermore, increased *fumC* expression caused by *S. aureus* results in lower levels of fumarate during infection of human macrophage-like cells (THP-1 cells) and peripheral blood mononuclear cells (PBMCs) (Raineri et al., 2022). *In vivo*, fumarate degradation by *S. aureus* results in diminished protection from a secondary staphylococcal challenge and promoted recurrent infection in a mouse model of skin infection (Raineri et al., 2022). *S. aureus* biofilms stimulate a metabolic bias in recruited macrophage and monocyte, favoring oxidative phosphorylation (OXPHOS) over glycolysis and facilitating their anti-inflammatory activity and biofilm persistence. These immune cells have anti-inflammatory properties including IL-10 and arginase production (Archer et al., 2011). Furthermore, it has been shown that exposure to fermentation supernatant of *S. aureus* results in chondrocyte degeneration, and further investigation indicates that, in response to fermentation supernatant of *S. aureus*, NF-κB signaling activation is coupled with increased cholesterol metabolism to stimulate catabolic factors in chondrocytes (Wang et al., 2022). However, the relationship between cholesterol metabolism and macrophages during *S. aureus* osteomyelitis remains uninvestigated.

Apolipoprotein E (ApoE) is a 35 kDa glycoprotein that belongs to a class of cellular proteins involved in lipid metabolism, which has an important role in cholesterol efflux and reverse cholesterol transport (Miao et al., 2023). Increasing studies have shown that ApoE is not only involved in cardiovascular disease, but also in degenerative disease, viral infection, and tumors (Tenger and Zhou, 2003; Zhu et al., 2012; Hui et al., 2022; Gao et al., n.d.). Based on these studies, it is worth investigating the role of ApoE in *S. aureus* osteomyelitis.

In the present study, we found that *S. aureus* osteomyelitis results in cholesterol loss from macrophage and alteration in intracellular cholesterol content affects bactericidal capacity of macrophage. We also identified ApoE as a core gene and ApoE deficiency attenuates *S. aureus* osteomyelitis infection both *in vivo* and *in vitro*. Mechanically, ApoE deficiency potentiates macrophage resistance to *S. aureus* osteomyelitis via regulating cholesterol metabolism. Our study reveals the role of ApoE in *S. aureus* osteomyelitis, suggesting that reprogramming of cholesterol metabolism is an essential host defense strategy against *S. aureus* osteomyelitis.

## 2 Materials and methods

### 2.1 Animals

C57BL/6 wild-type(WT)mice were purchased from the Experimental Animal Center, Southern Medical University (Guangzhou, China), and Ruisiyuan Biotechnology Co., Ltd (Zhaoqing, China). ApoE KO(*Apoe^-/-^*) mice were purchased from Ruisiyuan Biotechnology Co., Ltd (Zhaoqing, China). All mice were housed in facility with a 12 h light/dark cycle, 24 ± 2°C room temperature, and ad libitum access to water and food.

### 2.2 Preparation of bacteria

*S. aureus* was isolated from a patient with chronic osteomyelitis, and methicillin-sensitive *S. aureus* was identified using PHOENIX 100 (Becton, Dickinson Microbiology Systems, USA). A frozen stock of *S. aureus* strains was routinely grown on tryptic soy broth (TSB) with shaking at 180 rpm at 37°C for 16-18h and collected by centrifugation at 3,000 rpm for 10 min. The bacterial pellets were washed and resuspended in phosphate-buffered saline (PBS). The concentration of *S. aureus* was adjusted to an optical density (OD) of 0.5 at 600 nm, approximately equal to 1x10^8^ CFU/mL. According to different experimental requirements, *S. aureus* bacteria liquid was diluted to different concentrations.

### 2.3 Cell culture

A total of 5x10^5^ L929 cells were seeded in 50mL DMEM growth medium containing 10% fetal bovine serum in a 75 cm^2^ flask for 7 days without changing medium. After 7 days, the supernatant was collected as macrophage colony-stimulation factor.

For isolation of bone marrow derived macrophages (BMDMs), bone marrow cells were flushed out from tibias and femurs of 8–10-week-old C57BL/6 male mice. Erythrocytes were lysed by red blood cell lysing buffer (Cat.PH1594, Phygene) for 3 min at 4 °C. Next, the pellets were resuspended, after centrifugation, in BMDM growth medium (DMEM growth medium containing 10% fetal bovine serum and 25% L929 fibroblast supernatant) with 1% penicillin and streptomycin and incubated at 37°C and 5% CO2.

### 2.4 Filipin III staining

Filipin III (Cat.70440, Cayman) was dissolved in ethanol to reach a final concentration of 5 mg/mL. Cells were fixed with 4% paraformaldehyde (PFA) and stained with 50 mg/mL Filipin III for 30-min at room temperature. The emission and excitation of Filipin III was at 340-380 and 385-470nm, respectively. The images were captured with Zeiss LSM980 confocal microscope using a 40x objective.

### 2.5 Modified implant-associated osteomyelitis mouse model

To prepare infected implants, *S. aureus* bacteria liquid was diluted to 1 x 10^6^ CFU/mL. Sterile self-tapping screws (1.5 × 1.0 mm) were placed into the diluted bacteria liquid and shaken at 20 rpm for 10-15 min. Next, the self-tapping screws were transferred to an incubator at 37 °C for 10 min. After desiccation, the infected self-tapping screws were placed on ice for subsequent use. Prior to surgery, mice were anesthetized by intraperitoneal injection of tribromoethanol (125 mg/kg of body weight). After the right hind leg was shaved followed by disinfection with iodine, mice were placed in the supine position and a 5 mm incision was made on the lateral side of the leg. The third trochanter of femur was exposed by blunt separation of the muscles, and a canal was created by drilling distally into the marrow. Next, the infected self-tapping screw, described above, was drilled into the bone along the canal, with care not to penetrate the contralateral cortex of the femur. Finally, the incision was closed with a 5-0 suture. Protocols for animal experiments were approved by the Animal Care and Use Committee at Nanfang Hospital, Southern Medical University.

### 2.6 Flow sorting

By day 7 after infection, implanted femurs were harvested, and a single-cell suspension of bone marrow was prepared after passing through a 70-μm cell strainer (15-1070, Biologix). Red blood cells were lysed (Cat.PH1594, Phygene) before cells were counted. After being incubated with anti-mouse-CD16/CD32 (E-AB-F0997A, Elabscience) to block non-specific antibody staining, cells were incubated with a mixture of mouse-specific cell surface antibodies on ice, including anti-F4/80-PE (E-AB-F0995D, Elabscience) and anti-CD11b-APC (E-AB-F1081E, Elabscience). After two rounds of washing in fluorescence-activated cell sorting (FACS) buffer, samples were run on a flow cytometer (COULTER MoFlo XDP, BECKMAN, USA). The CD11b^+^F4/80^+^ cells were defined as macrophages and collected for further analysis.

### 2.7 Cholesterol measurement by LC-MS/MS based metabolomics approach

See **Supplementary Material** for a detailed description.

### 2.8 Phagocytosis assay

Mature BMDMs were detached by Accutase (Cat.00-4555-56, Invitrogen), centrifuged, and resuspended at 2×10^5^ cells/well in a 24-well tissue culture plate with antibiotic-free BMDM growth medium. For phagocytosis assay, the cells were incubated for 30 min at 37°C at a multiplicity of infection (MOI) of 10 using bacterial suspensions in PBS. After 30 min, BMDMs were washed using PBS, followed by lysis with Triton X-100 (0.1%). Lysis was serially diluted in PBS, and dilutions were spot-plated onto agar plate. After overnight incubation at 37°C, the CFUs of *S. aureus* were recorded as a measure of phagocytosis.

### 2.9 Intracellular killing assay

For intracellular killing assay, following infection for 30 min, BMDMs were washed and treated for 1 h with gentamicin (50 μg/mL) and lysozyme (2 μg/mL). After extracellular bactericidal process, BMDMs were cultured with a fresh medium containing antibiotics (1% penicillin/streptomycin) in the presence or absence of Simvastatin (HY-17502, MCE) or water-soluble cholesterol (HY-N0322A, MCE), or Avasimibe (HY-13215, MCE), or Terbinafine (HY-17395A, MCE). After 12 h treatment, the cells were lysed with 0.1% Triton X-100, and CFUs were counted to determine the intracellular killing rate.

### 2.10 Micro-computed tomography (micro-CT) imaging

Operated femurs were dissected free of soft tissue and self-tapping screw, fixed overnight in 4% paraformaldehyde, and analyzed by a high-resolution micro-CT (skyscan 1176, Bruker, Belgium). The scan was performed at an isotropic voxel size of 9 μm, a voltage of 50 kVp, a current of 200 μA and an integration time of 400 ms. Images were reconstructed and analyzed using software (NRecon, CTan, Bruker, Belgium). The region of interest (ROI) was defined as the 75-165 slices of proximal tibia bone starting from the growth plate. Structural parameters including bone volume fraction (BV/TV), bone mineral density (BMD), trabecular number (Tb.N), trabecular thickness (Tb.Th), and trabecular separation (Tb. Sp) were calculated.

### 2.11 Histological analysis

By day 7 after infection, mice were euthanized by cervical dislocation and perfused intracardially with 4% paraformaldehyde. The implanted femurs were harvested and fixed in 4% paraformaldehyde at 4°C overnight. After demineralization in 10% EDTA for 10 days, samples were processed and embedded in paraffin. Coronal sections of 4-μm thickness were cut and stained with hematoxylin and eosin. Smeltzer’s scoring methods (Smeltzer et al., 1997) were used to evaluate the histopathological changes by two blinded observers. Each section was assigned a score according to the sum of intraosseous acute inflammation (0–4), intraosseous chronic inflammation (0–4), periosteal inflammation (0–4), and bone necrosis (0–4). To detect bacterial burden in bone, Gram staining was performed on deparaffinized and rehydrated sections using a Gram Stain Kit (Modified Brown & Brenn) (Cat.BBS-2, Seytek).

### 2.12 Isolation of bone marrow and bone marrow supernatant

By day 7 after infection, mice were euthanized by cervical dislocation. The implanted femurs were harvested and the self-tapping screws were removed. The femurs were then placed in 0.5 ml bottomless microtubes that funneled into 1.5 ml Eppendorf microcentrifuge tubes. The nested tubes with bone were spun for 9 s at 13,000 x g to acquire bone marrow pellets. The pellets were resuspended in 450 μl red blood cell lysis buffer (Cat.PH1594, Phygene) and centrifuged at 500 × g for 3 min at 4°C. A volume of 400 μl of separated middle layer was collected as bone marrow supernatant for further analysis. The pellets were resuspended in 400 μl PBS and centrifuged at 500 x g for 3 min. After centrifugation, the pellets were collected for RNA-seq analysis.

### 2.13 Blood lipid measurement

A volume of 0.5 ml blood was collected from mice to extract serum, and serum total cholesterol (TC), serum triglycerides (TG), serum high-density lipoprotein cholesterol (HDL-C), serum low-density lipoprotein cholesterol (LDL-C), were determined by an automatic biochemical analyzer (BS330, Mindray, China).

### 2.14 RNA isolation and quantitative real-time PCR

Total RNA of BMDMs was extracted using RNA Purification kit (B0004DP, EZBioscience) according to manufacturer instructions. Reverse transcription into cDNA was performed using Evo Moloney Murine Leukemia Virus RT Premix (AG11706,Accurate Biology). Quantitative real-time PCR was performed using SYBR Green (AG11702, Accurate Biology) on QuantStudio5 (Applied Biosystems, USA) according to manufacturer protocol. The PCR primers are shown in (**Table S2**). The Beta-Actin genes were used as internal control. The relative amount of each gene was calculated using the 2^-ΔΔCT^ method.

### 2.15 RNA preparation and transcriptome sequencing

See **Supplementary Material** for a detailed description.

### 2.16 Differential expression analysis

The DESeq2 R package (1.22.1) was used for differential expression analysis. The resulting *P*-values were adjusted using Benjamini and Hochberg’s approach to control for false-discovery rate. For dataset GSE166522, genes with an adjusted *P*-value < 0.05 and | log2 (fold change) | value > 0.5 were recognized as differentially expressed genes (DEGs). For expression analysis between WT and *Apoe^-/-^* mice with or without *S. aureus* osteomyelitis, genes with an adjusted *P*-value < 0.05 and | log2 (fold change) | value >1 were recognized as DEGs.

### 2.17 Gene set enrichment analysis (GSEA) and leading-edge analysis

Cholesterol metabolism gene sets were selected through the Molecular Signatures Database (MsigDB). The collection of cholesterol metabolism gene sets was comprised of 93 gene sets (**Table S1**). The gene expression data of 14-day groups from GSE166522 was for GSEA, and the enriched gene sets (*P* < 0.01, FDR < 0.25) on *S. aureus* osteomyelitis group were continued to perform a leading-edge analysis. All analyses were performed using GSEA software (version 4.2.3).

### 2.18 Statistical analysis

Statistical analysis was performed using SPSS 26.0 software (IBM SPSS 26.0, SPSS Inc.). For comparisons between two groups (parametric data), Student’s t-test was applied. Mann-Whitney U-test was used for nonparametric data. For multigroup comparisons, one-way analysis of variance (ANOVA), with LSD (equal variance assumed) or Dunnett T3 test (equal variance not assumed), was used. All data are expressed as mean ± SEM.

## 3 Result

### 3.1 *S. aureus* induces loss of cholesterol in macrophages in the infected-bone of mice

**Figure 1:**
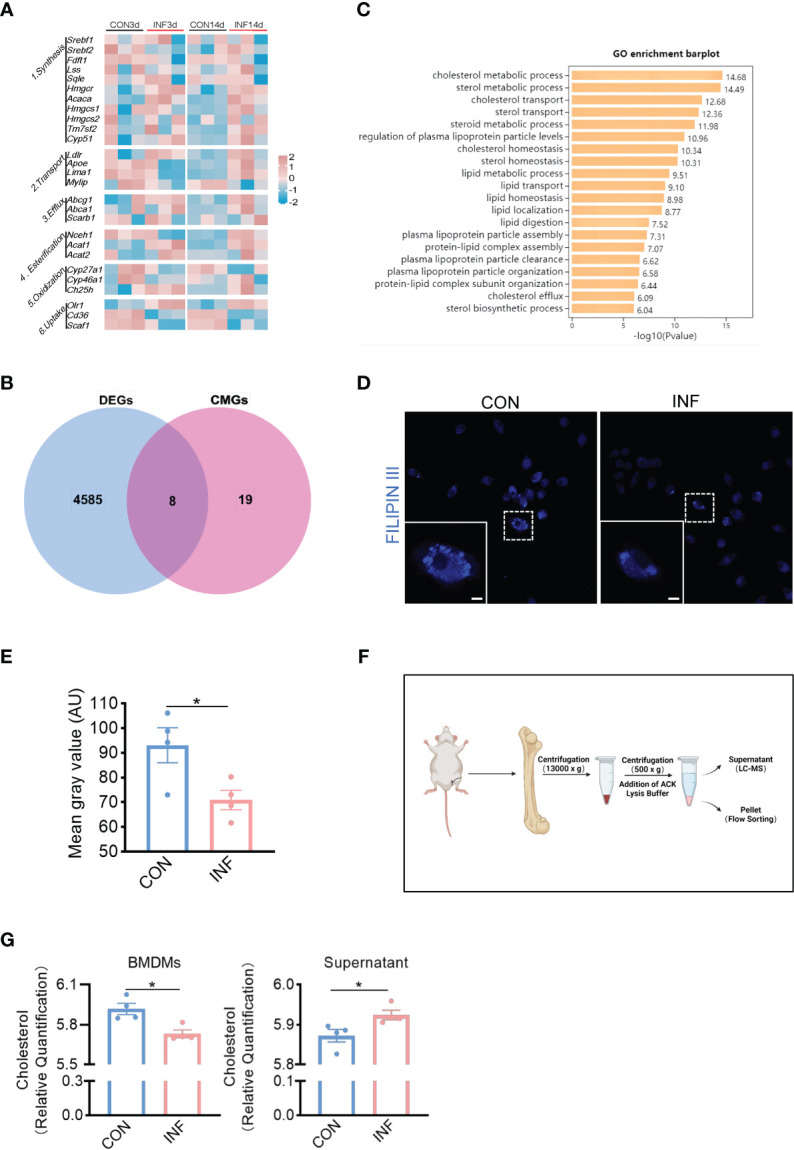
*S. aureus* induces loss of cholesterol in macrophages in the infected-bone of mice. **(A)** Heatmap showing relative expression of CMGs in *S. aureus* infected femur on day 3 and 14 post-infection. **(B)** Venn diagram showing number of differentially expressed CMGs on day 14 post-infection. **(C)** GO enrichment analysis of differentially expressed CMGs on day 14 post-infection. **(D)** Representative images of Filipin III staining in BMDMs infected with S. aureus for 12 hours. Scale bar was 5μm. **(E)** Fluorescence intensity result of Filipin III staining. **(F)** Schematic of the isolation of bone marrow and bone marrow supernatant. Images were created with BioRender.com. **(G)** Cholesterol content in CD11b^+^F4/80^+^ macrophages and supernatant from *S. aureus* osteomyelitis mice and controls. n = 4/group. Data are presented as mean ± SEM. Two-tailed Student’s t-test was used. ∗*P* < 0.05.

In order to determine whether cholesterol metabolism was affected in the bone of *S. aureus* osteomyelitis mice, we analyzed the expression of cholesterol metabolism genes (CMGs) (Xiao et al., 2020) in femur with *S. aureus* osteomyelitis from GSE166522 (Lin et al., 2021). Compared to CMGs by day 3 post-infection, the differential expression of CMGs by day 14 post-infection was more pronounced (**Figure 1A**). Differentially expressed CMGs were chosen among differentially expressed genes by day 14 post-infection, followed by GO (Gene Ontology) enrichment analysis (**Figure 1B**). The differentially expressed CMGs were significantly enriched in a variety of cholesterol metabolism pathways (**Figure 1C**), indicating cholesterol metabolism might be altered during the pathogenesis of *S. aureus* osteomyelitis.

We then sought to determine the relationship between cholesterol content of macrophages and *S. aureus* osteomyelitis. The Filipin III results showed that *S. aureus* infection of BMDMs for 12 hours resulted in cholesterol loss (**Figures 1D, E**). Before detecting changes in the cholesterol content of BMDMs *in vivo*, we modified the establishment of mice model with *S. aureus* osteomyelitis on the basis of a previous method (Lin et al., 2021) (**Figure S1A**), and the histochemical staining results showed that the modified protocol was practicable and stable (**Figures S1B,C**). The modified protocol was similar to the clinical way of internal fixation. Moreover, it eliminated the injection of *S. aureus* during establishment and facilitated the removal of implant. Subsequently, we isolated the bone marrow macrophages and bone marrow supernatant from mice with S. aureus osteomyelitis by day 7 after operation (**Figure 1F**). Using a liquid chromatography-tandem mass spectrometry (LC-MS/MS)–based metabolomics approach, we assessed the impact of S. aureus osteomyelitis on cholesterol content of CD11b+F4/80+ macrophages and supernatant. S. aureus osteomyelitis led to decreased population of CD11b+F4/80+ macrophages (**Figure S2A**), increased cholesterol content of supernatant, and reduced cholesterol content of CD11b+F4/80+ macrophages (**Figure 1G**). Together, these data suggest that S. aureus osteomyelitis induces cholesterol content modulation in infected femur, which leads to diminished cholesterol content of infected macrophages.

### 3.2 ApoE is the key factor in cholesterol metabolism disorder during *S. aureus* osteomyelitis

**Figure 2:**
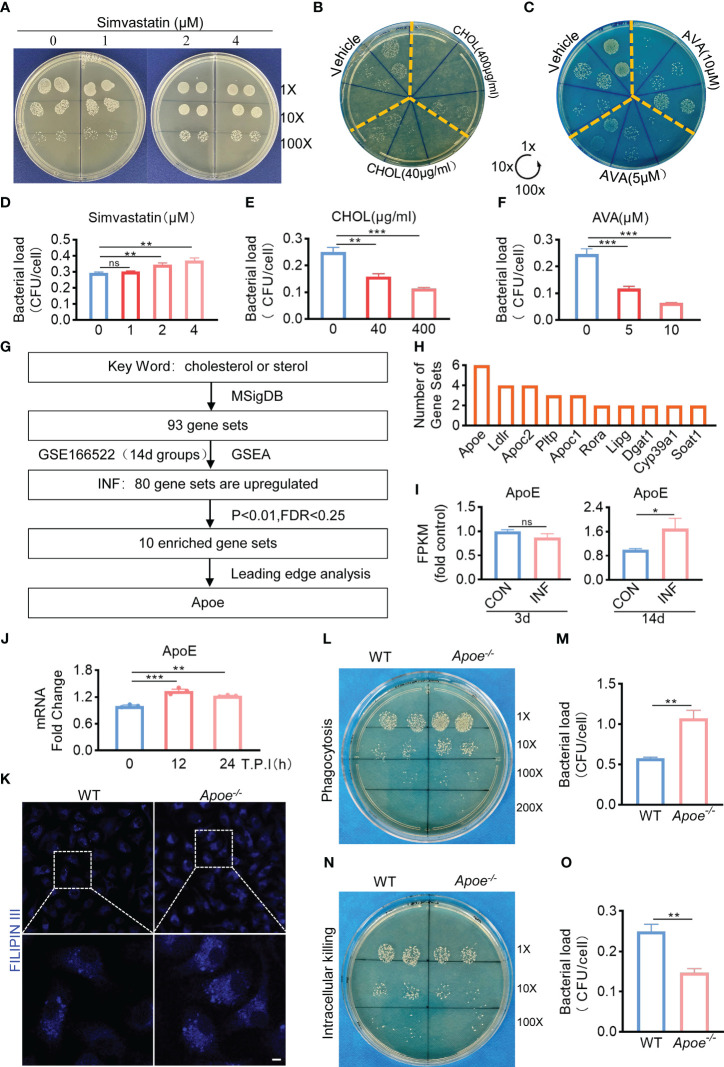
ApoE is the key factor in cholesterol metabolism disorder during *S. aureus* osteomyelitis. **(A-F)** Representative images **(A–C)** and quantification **(D– F)** of CFUs of *S. aureus* for the bactericidal assay. BMDMs were infected with *S. aureus* (MOI = 10) for 30 min. After extracellular bacteria were removed, cells were treated with Simvastatin, water-soluble cholesterol (CHOL), Avasimibe (AVA) for 12 h. n = 3/group. **(G)** Flowchart of collection of cholesterol metabolism gene sets, gene set enrichment analysis, screening for significantly enriched gene sets and leading-edge analysis. **(H)** The result of leading-edge analysis. **(I)** Normalized gene expression of ApoE in mice at day 3 or 14 after *S. aureus* osteomyelitis from GSE166522.n=3/group. **(J)** Quantification of mRNA expression of ApoE in BMDMs infected with *S. aureus* at an MOI of 10 for 12h and 24h. n = 3/group. **(K)** Representative images of Filipin III staining in WT or *Apoe* ^-/-^ BMDMs. Scale bar was 10μm. **(L, M)** Representative images **(L)** and quantification **(M)** of CFUs of *S. aureus* for the phagocytosis assay. WT or *Apoe* ^-/-^ BMDMs were infected with *S. aureus* (MOI = 10) for 30 min. n = 3/group. **(N, O)** Representative images **(N)** and quantification **(O)** of CFUs of *S. aureus* for the bactericidal assay. WT or *Apoe* ^-/-^ BMDMs were infected with *S. aureus* (MOI = 10) for 30 min. After extracellular bacteria were removed, cells were treated for 12 h. n = 3/group. Data are presented as mean ± SEM. One-way ANOVA with Tukey’s test **(D-F, J)** and two-tailed Student’s t-test **(I, M, O)** were used. *:*P <* 0.05, **:*P <* 0.01, ***:*P <* 0.001. "ns" means "not statistically significant".

Based on the results mentioned above, we hypothesized that the amount of intracellular cholesterol could influence the killing function of macrophages. We used Simvastatin, a competitive inhibitor of HMG-CoA reductase, that prevents cholesterol biosynthesis (Corsini et al., 1999). Comparing to control, Simvastatin significantly reduces the killing potency of infected BMDMs (**Figures 2A, B**). On the other hand, bactericidal function of BMDMs was upregulated under the treatment with water-soluble cholesterol (**Figures 2C, D**). In addition, we used Avasimibe, an acyl coenzyme A-cholesterol acyltransferase inhibitor, that relatively enhances cholesterol content via blocking the conversion of cholesterol to cholesterol esterase (Ohshiro et al., 2011). We noted that Avasimibe treatment enhance bactericidal activity of BMDMs upon *S. aureus* infection (**Figures 2E, F**). Overall, these findings indicate that regulating intracellular cholesterol could alter bactericidal capacity of macrophages against *S. aureus*.

Furthermore, to validate the key factor in cholesterol metabolism disorder during *S. aureus* osteomyelitis, the gene expression data of 14-day groups from GSE166522 was for GSEA, and we continued to perform a leading-edge analysis of the significantly enriched gene sets (*P* < 0.01, FDR < 0.25) on *S. aureus* osteomyelitis group, identifying the core driver (**Figure 2G**). The result of leading-edge analysis showed that ApoE was associated with the largest number of enriched gene sets (**Figure 2H**), and we found that expression of ApoE increased by day 14 post-infection, despite expression levels of ApoE comparable to control by day 3 post-infection (**Figure 2I**). We therefore assessed the expression of ApoE in BMDMs after stimulation of *S. aureus in vitro*. The qPCR results revealed, compared to control, a small, but significant, elevation of mRNA expression of ApoE in infected BMDMs (**Figure 2J**). We then isolated BMDMs from ApoE knockout (*Apoe^-/-^*) mice to examine the impact of ApoE deficiency on the function of macrophages in response to *S. aureus* infection. The ApoE deficiency led to increased cholesterol content (**Figure 2K**), as well as enhanced phagocytosis and bactericidal rate of BMDMs (**Figures 2L–O**), indicating that ApoE deficiency may affect innate immune functions of macrophages via regulation of cholesterol metabolism.

### 3.3 ApoE deficiency ameliorates bacterial burden and bone destruction in mice with *S. aureus* osteomyelitis

**Figure 3:**
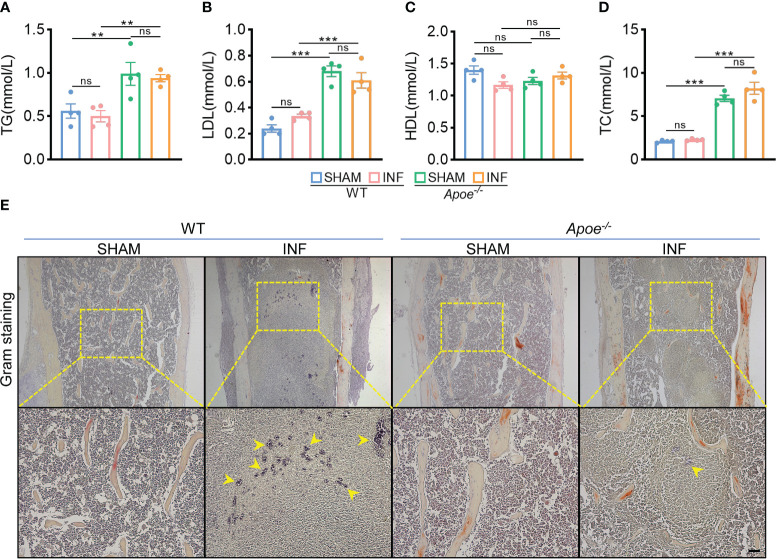
ApoE deficiency mitigates bacterial load in mice with *S. aureus* osteomyelitis. **(A–D)** Total triglyceride **(A)**, low-density lipoprotein **(B)**, high-density lipoprotein **(C)**, and total cholesterol content **(D)** of WT or *Apoe* ^-/-^ mice with or without *S. aureus* osteomyelitis. n = 4/group. **(E)** Representative images of gram staining of femurs in WT or *Apoe* ^-/-^ mice with or without *S. aureus* osteomyelitis. Yellow arrows indicate *S. aureus.* Scale bar was 100um.Data are presented as mean ± SEM. One-way ANOVA with Tukey’s test were used. **:*P <* 0.01, ***:*P <* 0.001. “ns” means “not statistically significant”.

**Figure 4:**
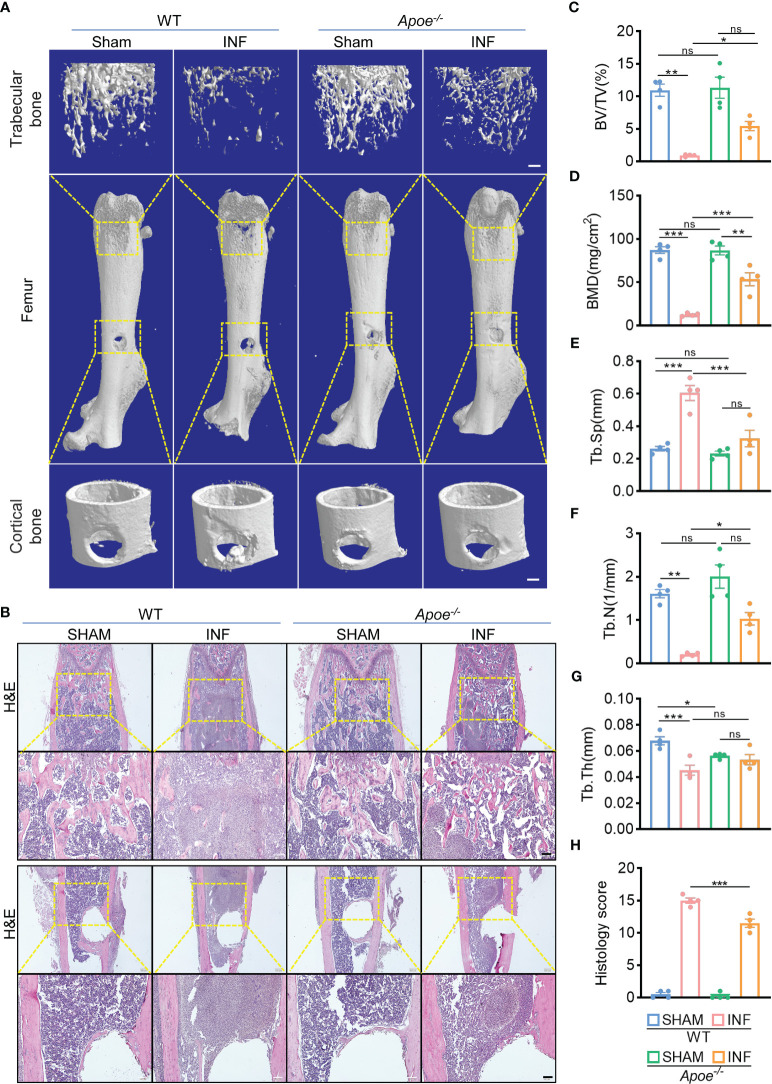
ApoE deficiency alleviates bone destruction in mice with *S. aureus* osteomyelitis. **(A)** Representative 3D images of femurs in WT or *Apoe* ^-/-^ mice with or without *S. aureus* osteomyelitis. Scale bar was 200um. **(B)** Representative images of H&E staining of femurs in WT or *Apoe* ^-/-^ mice with or without *S. aureus* osteomyelitis. Scale bar was 100um. **(C–G)** Quantitative analysis of trabecular bone fraction (BV/TV) **(C)**, bone mineral density (BMD) **(D)**, trabecular separation (Tb. Sp) **(E)**, trabecular number (Tb. N) **(F)**, and trabecular thickness (Tb.Th) **(G)** of the femur from WT or *Apoe* ^-/-^ mice with or without *S. aureus* osteomyelitis. n = 4/group. **(H)** Quantitative analysis of histopathological changes using Smeltzer’s scoring method. n = 4/group. Data are presented as mean ± SEM. One-way ANOVA with Tukey’s test were used. *:*P* < 0.05, **:*P* < 0.01, ***:*P* < 0.001. “ns” means “not statistically significant”.

To confirm the role of blocking ApoE against *S. aureus* osteomyelitis, we then developed *S. aureus* osteomyelitis models in wild-type (WT) mice and ApoE knockout (*Apoe^-/-^*) mice. Before the establishment of mice models with *S. aureus* osteomyelitis, we confirmed that ApoE deficiency has no significant effect on the bone mass of 8-week-old male mice fed normal chow (**Figure S3A**). By day 7 after operation, several lipid parameters indicated that, though ApoE deficiency elevated total cholesterol (TC), total triglyceride (TG), and low-density lipoprotein (LDL) contents in *Apoe* ^-/-^ mice, *S. aureus* had no significant effect on lipid profile of infected-femurs of mice regardless of the presence or absence of ApoE (**Figures 3A–D**). We observed a considerable amount of Gram staining in the bone marrow of WT mice with *S. aureus* osteomyelitis, whereas sparse Gram staining could be noted in *Apoe* ^-/-^ mice (**Figure 3E**). Micro-CT data confirmed the protective role of ApoE deficiency against *S. aureus*-induced of bone destruction. *Apoe^-/-^* mice showed improved trabecular bone in the distal femur and reduced cortical bone loss in the implant area compared with WT mice (**Figure 4A**). The higher bone volume fraction (BV/TV) and bone mineral density (BMD) was associated with an increased trabecular number (Tb.N) and reduced trabecular separation (Tb.Sp) (**Figures 4C–G**). Consistent with the micro-CT results, histological staining and scores showed smaller areas of abscesses and decreased bone destruction in *Apoe* ^-/-^ mice relative to WT mice (**Figures 4B, H**). These data suggest that blocking ApoE might promote bacterial clearance and hinder bone destruction.

### 3.4 ApoE deficiency mediates macrophage resistance to *S. aureus* osteomyelitis via regulation of cholesterol metabolism.

**Figure 5:**
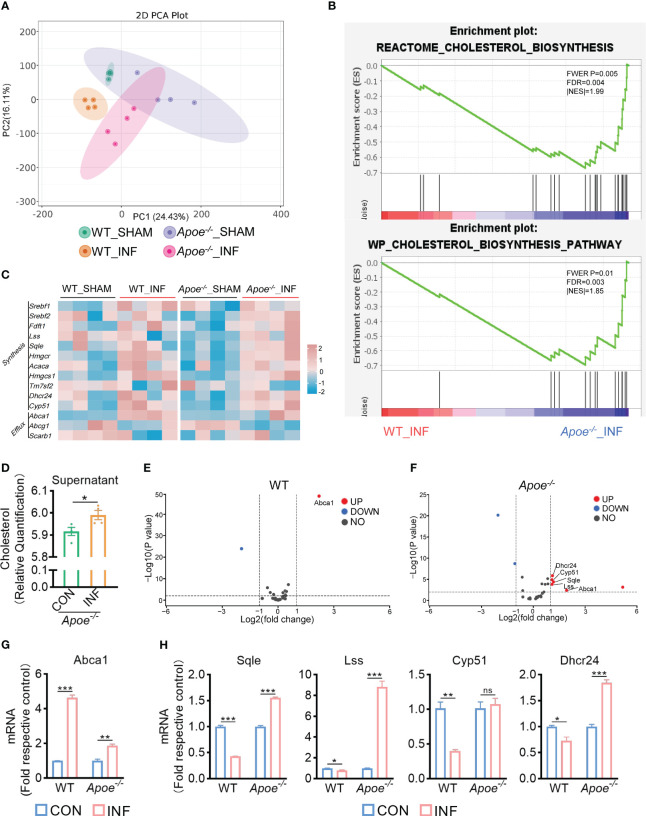
ApoE deficiency enhances cholesterol biosynthesis after *S. aureus* osteomyelitis. **(A)** Principal component analysis result. n = 4/group. **(B)** GSEA results showing the cholesterol biosynthesis pathway significantly enriched in *Apoe* ^-/-^ mice compared with WT mice, under *S. aureus* osteomyelitis conditions. **(C)** Heatmap showing relative expression of cholesterol efflux and cholesterol biosynthesis genes in femur bone marrow of WT and *Apoe* ^-/-^ mice with or without *S. aureus* osteomyelitis. **(D)** Cholesterol content in supernatant from *Apoe* ^-/-^ mice with or without *S. aureus* osteomyelitis. n = 4/group. **(E)** Volcano plot showing relative expression of CMGs in femur bone marrow of WT mice with or without *S. aureus* osteomyelitis. **(F)** Volcano plot showing relative expression of CMGs in femur bone marrow of *Apoe* ^-/-^ mice with or without *S. aureus* osteomyelitis. **(G)** mRNA expression of ABCA1 of WT or *Apoe* ^-/-^ BMDMs stimulated by *S. aureus* for 6h. n = 3/group. **(H)** mRNA expression of SQLE, LSS, CYP51, and DHCR24 of WT or *Apoe* ^-/-^ BMDMs stimulated by *S. aureus* for 12h. n = 3/group. Data are presented as mean ± SEM. One-way ANOVA with Tukey’s test **(G, H)** and two-tailed Student’s t-test **(D)** were used. *:*P <* 0.05, **:*P <* 0.01, ***:*P <* 0.001. “ns” means “not statistically significant”.

**Figure 6:**
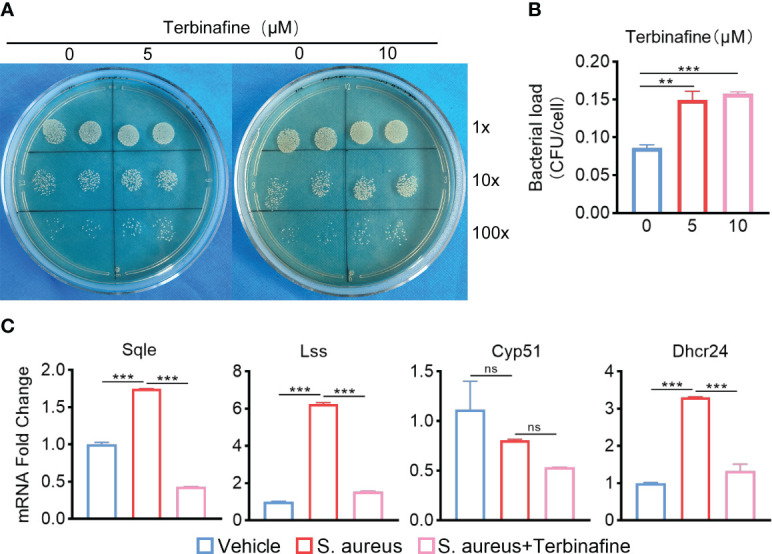
Terbinafine impairs the bactericidal capacity of *Apoe* ^-/-^ BMDMs via blocking cholesterol biosynthesis. **(A, B)** Representative images **(A)** and quantification **(B)** of CFUs of *S. aureus* for the bactericidal assay. *Apoe* ^-/-^ BMDMs were infected with *S. aureus* (MOI = 10) for 30 min. After extracellular bacteria were removed, cells were treated with Terbinafine for 12 h. n = 3/group. **(C)** mRNA expression of SQLE, LSS, CYP51, and DHCR24 of *Apoe* ^-/-^ BMDMs stimulated by *S. aureus* for 12h, in the presence or absence of Terbinafine (10μM). n = 3/group. Data are presented as mean ± SEM. One-way ANOVA with Tukey’s test were used. **:*P <* 0.01, ***:*P <* 0.001. “ns” means “not statistically significant”.

To explore how ApoE functions in the regulation of cholesterol metabolism during *S. aureus* osteomyelitis, we performed transcriptome analysis of the WT and *Apoe* ^-/-^ femur bone marrow using high-throughput sequencing. The principal component analysis result showed that ApoE deficiency caused some degree of gene expression variation between WT and *Apoe* ^-/-^ mice, but the effect of *S. aureus* infection on gene expression dispersion was more significant, suggesting, at the transcriptional level, ApoE affects the outcome of *S. aureus* osteomyelitis (**Figure 5A**). Because previous studies reported that ApoE is related to cholesterol efflux (Getz and Reardon, 2018), we conjectured that ApoE may influence cholesterol efflux in infected macrophages. However, the GSEA results showed that cholesterol biosynthesis gene sets were strongly enriched in *Apoe^-/-^* mice with *S. aureus* osteomyelitis (**Figure 5B**). Beyond this, bone marrow supernatant from mice with *S. aureus* osteomyelitis had an elevated level of cholesterol content and an upregulated mRNA expression of ATP-binding cassette transporter A1 (ABCA1), a critical transporter for cholesterol efflux, was detected in *S. aureus*-infected BMDMs,independent of the presence of the ApoE (**Figures 5C–G**). On the other hand, the expression of cholesterol biosynthesis genes showed a significant increase in *Apoe^-/-^* mice with *S. aureus* osteomyelitis, including Squalene Epoxidase (SQLE), Lanosterol Synthase (LSS), Cytochrome P450 Family 51 Subfamily (CYP51), 24-Dehydrocholesterol Reductase (DHCR24) (**Figures 5C, F**). We then detected the expression of these genes in BMDMs *in vitro*, and qPCR results were consistent with transcriptome analysis (**Figure 5H**). We observed that these upregulated cholesterol biosynthesis genes are in the pathway form squalene to lanosterol and assumed that killing capacity of *Apoe* ^-/-^ BMDMs would be impaired via blocking this pathway. We used SQLE inhibitor, Terbinafine, that prevents the conversion of squalene to lanosterol (Nowosielski et al., 2011). In comparison to control, the killing ability of ApoE-deleted BMDMs was reduced under Terbinafine treatment (**Figures 6A, B**), as well as induction of the cholesterol biosynthesis genes SQLE, LSS, and DHCR24 (**Figure 6C**). Overall, these findings indicate that the mechanism behind ApoE deficiency ameliorating macrophage resistance to *S. aureus* osteomyelitis is not only through augmentation of intracellular cholesterol caused by ApoE knockout, but also through reducing cholesterol loss via enhanced cholesterol biosynthesis.

## 4 Discussion

Cholesterol, a fat-like substance, is the main steroidal compound in mammals and plays a vital role in basic cellular life activities. Some bacterial pathogens rely on the host cell for a sizable percentage of their growth requirements and possess sophisticated mechanisms to manipulate the host cell to obtain essential nutrients. One of the targeted host cell factors is cholesterol. Pathogenic bacteria target cholesterol not only to gain entry to host cells, but also to hijack host cell signaling pathways favorable for intracellular survival (Samanta et al., 2017). *Chlamydia pneumoniae* (*C. pneumoniae*) infection decreased cholesterol efflux by downregulating expression of ABCA1 in A549 lung epithelial cell lines (Korhonen et al., 2013). Experimental measurement of cholesterol efflux to ApoA-1 showed a 50% decrease in *C. pneumoniae*-infected THP-1 macrophage-like foam cells compared to uninfected or heat-killed bacteria-infected cells. Further, *C. pneumoniae* appeared to downregulate host cholesterol efflux by increasing microRNA miR-33 levels, which is produced from the SREBP intron, and downregulates ABCA1 (Zhao et al., 2014). *Coxiella burnetii* (*C. burnetii*) differentially regulated ApoE and APOC gene expression in THP-1 macrophages (Ren et al., 2003). In addition, a genome-wide RNA interference screen in HeLa cells revealed that siRNA depletion of apolipoproteins involved in lipid transport, affected the total number of *C. burnetii* parasitophorous vacuoles (McDonough et al., 2013). Although research on the association between *S. aureus* and cholesterol metabolism of macrophages is limited, previous study reported that fermentation supernatant of *S. aureus* resulted in chondrocyte degeneration through NF-κB signaling activation coupled with increased cholesterol metabolism, stimulating catabolic factors in chondrocytes (Q et al., 2022). Our data show the enhanced cholesterol efflux and subsequent cholesterol loss in bone marrow macrophages of mice with *S. aureus* osteomyelitis. Moreover, cholesterol is associated with inflammation, polarization, and mitochondrial-derived reactive oxygen species(mtROS) (Sheedy et al., 2013; O’Rourke et al., 2022), which are critical for anti-bacterial activity of macrophages (West et al., 2011; Galli and Saleh, 2021).We assume that cholesterol loss of macrophage induced by *S. aureus* infection could impair bactericidal capacity of macrophages via the switch of macrophages toward M2 polarization or decreased generation of mtROS, promoting *S. aureus* survival. Thus, modulation of cholesterol metabolism in macrophages might be one way to alleviate *S. aureus* osteomyelitis, based on our present data.

ApoE was initially described as a lipid transport protein and major ligand for LDL receptors with a role in cholesterol metabolism and cardiovascular disease. ApoE deficiency is associated with decreased catabolism of atherogenic lipoproteins, favoring hypercholesterolemia and atherosclerosis development (Zhang et al., 1992). Recent studies showed that ApoE is involved in the pathogenesis of infection and tumor development. For example, ApoE is required for infectious viral particle production of Hepatitis C virus (HCV) (Gong and Cun, 2019). ApoE KO mice exhibit increased susceptibility to influenza A virus infection and severe disease pathology than WT mice, because ApoE-deleted cells exhibit an increased surface distribution of IAV receptor sialic acid via increased membrane cholesterol (Gao et al., 2022). In contrast, ApoE KO mice had significantly lower herpes simplex virus-1 concentrations in the nervous system than WT mice (Burgos et al., 2006). Additionally, ApoE inhibits HIV infection by directly interacting with HIV gp160 and suppresses Env expression (Siddiqui et al., 2018). On the other hand, single-cell transcriptomics of cholangiocarcinoma and anti-CSF1R–treated tumors, identified a unique granulocytic myeloid-derived suppressor cell (G-MDSCs) subset, ApoE G-MDSCs, with abundant expression of ApoE in the vehicle-treated tumors and marked ApoE downregulation with tumor-associated tumor blockade (Loeuillard et al., 2020). ApoE activation has been linked to enhanced MDSC apoptosis and consequent tumor regression (Tavazoie et al., 2018). Moreover, *Apoe* ^-/-^ mice exhibited higher resistance toward the development of three types of carcinomas as compared to wild-type mice and had greater responses to αPD-1 (anti-PD-1) immunotherapy (Hui et al., 2022). Our observation that ApoE knockout enhances innate immune function of BMDMs and ameliorates bone infection and destruction in mice with *S. aureus* osteomyelitis. Further transcriptome analyses and *in vitro* experiments evaluate how ApoE deficiency enhances cholesterol biosynthesis to inhibit cholesterol loss, mediating macrophage resistance to *S. aureus* osteomyelitis. Though ApoE is mostly linked to cholesterol efflux and reverse cholesterol transport, it is reported that astrocytic ApoE vectors a variety of microRNAs (miRNAs) that specifically silence genes involved in neuronal cholesterol biosynthesis (Li et al., 2021). However, we cannot exclude the possibility that ApoE may affect the pathogenesis of *S. aureus* osteomyelitis through other mechanisms.

Although we found that ApoE deficiency could improve macrophage bacterial clearance in *S. aureus* osteomyelitis, there are some limitations in our study. First, we did not use myeloid specific ApoE knockout mice to further verify the mechanism of ApoE action in macrophages. Second, it remains to be determined how *S. aureus* may induce activation of ApoE in bone marrow macrophages, as well as the downstream mechanisms by which alteration in intracellular cholesterol content affects bactericidal capacity of macrophages. Third, whether ApoE mediates the suppressed bactericidal activity of macrophages against other bacterial species deserves further investigation.

In conclusion, our data demonstrate mice bone with *S. aureus* osteomyelitis has a reduced level of cholesterol in macrophages, and regulation of cholesterol metabolism could alter the bactericidal capacity of macrophages. We also identified ApoE as a key factor and established that ApoE deficiency functions as a protective factor against *S. aureus* osteomyelitis via regulating cellular cholesterol metabolism.

## Data availability statement

The datasets presented in this study can be found in online repositories. The names of the repository/repositories and accession number(s) can be found below: https://www.ncbi.nlm.nih.gov/geo/ , GSE227521.

## Ethics statement

The animal study was reviewed and approved by The Animal Care and Use Committee at Nanfang Hospital, Southern Medical University.

## Author contributions

Conceptualization, BY, XZ, and ML. Bioinformatic analysis, ML and CL. Mouse experiments, ML, RH, CL, ZL, YC, and BSY. *In vitro* experiments, ML, CL, and ZL. Statistical analysis, ML and RH. Original draft, ML. Figure preparation, RH and ML. Critical revision of the manuscript, XZ. All authors contributed to the article and approved the submitted version.

## References

[B1] Acharya S. Soliman M. Egun A. Rajbhandari S. M. (2013). Conservative management of diabetic foot osteomyelitis. Diabetes Res. Clin. Pract. 101, e18–e20. doi: 10.1016/j.diabres.2013.06.010 23850116

[B2] Archer N. K. Mazaitis M. J. Costerton J. W. Leid J. G. Powers M. E. Shirtliff M. E. (2011). Staphylococcus aureus biofilms: properties, regulation, and roles in human disease. Virulence. 2, 445–459. doi: 10.4161/viru.2.5.17724 21921685 PMC3322633

[B3] Berends E. T. M. Zheng X. Zwack E. E. Ménager M. M. Cammer M. Shopsin B.. (2019). Staphylococcus aureus impairs the function of and kills human dendritic cells via the LukAB toxin. mBio. 10, e01918–e01918. doi: 10.1128/mBio.01918-18 30602580 PMC6315100

[B4] Burgos J. S. Ramirez C. Sastre I. Valdivieso F. (2006). Effect of apolipoprotein e on the cerebral load of latent herpes simplex virus type 1 DNA. J. Virol. 80, 5383–5387. doi: 10.1128/JVI.00006-06 16699018 PMC1472141

[B5] Corsini A. Bellosta S. Baetta R. Fumagalli R. Paoletti R. Bernini F. (1999). New insights into the pharmacodynamic and pharmacokinetic properties of statins. Pharmacol. Ther. 84, 413–428. doi: 10.1016/S0163-7258(99)00045-5 10665838

[B6] Galli G. Saleh M. (2021). Immunometabolism of macrophages in bacterial infections. Front. Cell Infect. Microbiol. 10. doi: 10.3389/fcimb.2020.607650 PMC7879570 33585278

[B7] Gao P. Ji M. Liu X. Chen X. Liu H. Li S.. (2022). Apolipoprotein e mediates cell resistance to influenza virus infection. Sci. Adv. 8, eabm6668. doi: 10.1126/sciadv.abm6668 36129973 PMC9491715

[B8] Getz G. S. Reardon C. A. (2018). Apoprotein e and reverse cholesterol transport. Int. J. Mol. Sci. 19, 3479. doi: 10.3390/ijms19113479 30404132 PMC6275009

[B9] Gong Y. Cun W. (2019). The role of ApoE in HCV infection and comorbidity. Int. J. Mol. Sci. 20, 2037. doi: 10.3390/ijms20082037 31027190 PMC6515466

[B10] Hubler M. J. Kennedy A. J. (2016). Role of lipids in the metabolism and activation of immune cells. J. Nutr. Biochem. 34, 1–7. doi: 10.1016/j.jnutbio.2015.11.002 27424223 PMC5694687

[B11] Hui B. Lu C. Li H. Hao X. Liu H. Zhuo D.. (2022). Inhibition of APOE potentiates immune checkpoint therapy for cancer. Int. J. Biol. Sci. 18, 5230–5240. doi: 10.7150/ijbs.70117 36147474 PMC9461658

[B12] Korhonen J. T. Olkkonen V. M. Lahesmaa R. Puolakkainen M. (2013). ABC-Cassette transporter 1 (ABCA1) expression in epithelial cells in chlamydia pneumoniae infection. Microbial Pathogenesis. 61–62, 57–61. doi: 10.1016/j.micpath.2013.05.006 23707398

[B13] Li X. Zhang J. Li D. He C. He K. Xue T.. (2021). Astrocytic ApoE reprograms neuronal cholesterol metabolism and histone-acetylation-mediated memory. Neuron. 109, 957–970.e8. doi: 10.1016/j.neuron.2021.01.005 33503410

[B14] Lin Y. Su J. Wang Y. Xu D. Zhang X. Yu B. (2021). mRNA transcriptome analysis of bone in a mouse model of implant-associated staphylococcus aureus osteomyelitis. Infect. Immun. 89, e00814–e00820. doi: 10.1128/IAI.00814-20 33619031 PMC8091086

[B15] Loeuillard E. Yang J. Buckarma E. Wang J. Liu Y. Conboy C.. (2020). Targeting tumor-associated macrophages and granulocytic myeloid-derived suppressor cells augments PD-1 blockade in cholangiocarcinoma. J. Clin. Invest. 130, 5380–5396. doi: 10.1172/JCI137110 32663198 PMC7524481

[B16] Masters E. A. Ricciardi B. F. Bentley K. L. de M. Moriarty T. F. Schwarz E. M.. (2022). Skeletal infections: microbial pathogenesis, immunity and clinical management. Nat. Rev. Microbiol. 20, 385–400. doi: 10.1038/s41579-022-00686-0 35169289 PMC8852989

[B17] McDonough J. A. Newton H. J. Klum S. Swiss R. Agaisse H. Roy C. R. (2013). Host pathways important for coxiella burnetii infection revealed by genome-wide RNA interference screening. mBio. 4, e00606–e00612. doi: 10.1128/mBio.00606-12 23362322 PMC3560531

[B18] Metsemakers W. J. Kuehl R. Moriarty T. F. Richards R. G. Verhofstad M. H. J. Borens O.. (2018). Infection after fracture fixation: current surgical and microbiological concepts. Injury. 49, 511–522. doi: 10.1016/j.injury.2016.09.019 27639601

[B19] Miao G. Zhuo D. Han X. Yao W. Liu C. Liu H.. (2023). From degenerative disease to malignant tumors: insight to the function of ApoE. Biomedicine Pharmacotherapy. 158, 114127. doi: 10.1016/j.biopha.2022.114127 36516696

[B20] Nowosielski M. Hoffmann M. Wyrwicz L. S. Stepniak P. Plewczynski D. M. Lazniewski M.. (2011). Detailed mechanism of squalene epoxidase inhibition by terbinafine. J. Chem. Inf Model. 51, 455–462. doi: 10.1021/ci100403b 21229992

[B21] Ohshiro T. Matsuda D. Sakai K. Degirolamo C. Yagyu H. Rudel L. L.. (2011). Pyripyropene a, an acyl–coenzyme a: cholesterol acyltransferase 2–selective inhibitor, attenuates hypercholesterolemia and atherosclerosis in murine models of hyperlipidemia. ATVB. 31, 1108–1115. doi: 10.1161/ATVBAHA.111.223552 21393580

[B22] O’Rourke S. A. Neto N. G. B. Devilly E. Shanley L. C. Fitzgerald H. K. Monaghan M. G.. (2022). Cholesterol crystals drive metabolic reprogramming and M1 macrophage polarisation in primary human macrophages. Atherosclerosis. 352, 35–45. doi: 10.1016/j.atherosclerosis.2022.05.015 35667162

[B23] Ozinsky A. Underhill D. M. Fontenot J. D. Hajjar A. M. Smith K. D. Wilson C. B.. (2000). The repertoire for pattern recognition of pathogens by the innate immune system is defined by cooperation between toll-like receptors. Proc. Natl. Acad. Sci. U S A. 97, 13766–13771. doi: 10.1073/pnas.250476497 11095740 PMC17650

[B24] Potter A. D. Butrico C. E. Ford C. A. Curry J. M. Trenary I. A. Tummarakota S. S.. (2020). Host nutrient milieu drives an essential role for aspartate biosynthesis during invasive staphylococcus aureus infection. Proc. Natl. Acad. Sci. U S A. 117, 12394–12401. doi: 10.1073/pnas.1922211117 32414924 PMC7275739

[B25] Raineri E. J. M. Altulea D. van Dijl J. M. (2022). Staphylococcal trafficking and infection-from “nose to gut” and back. FEMS Microbiol. Rev. 46, fuab041. doi: 10.1093/femsre/fuab041 34259843 PMC8767451

[B26] Ren Q. Robertson S. J. Howe D. Barrows L. F. Heinzen R. A. (2003). Comparative DNA microarray analysis of host cell transcriptional responses to infection by coxiella burnetii or chlamydia trachomatis. Ann. N Y Acad. Sci. 990, 701–713. doi: 10.1111/j.1749-6632.2003.tb07447.x 12860710

[B27] Russell D. G. Huang L. VanderVen B. C. (2019). Immunometabolism at the interface between macrophages and pathogens. Nat. Rev. Immunol. 19, 291–304. doi: 10.1038/s41577-019-0124-9 30679807 PMC7032560

[B28] Samanta D. Mulye M. Clemente T. M. Justis A. V. Gilk S. D. (2017). Manipulation of host cholesterol by obligate intracellular bacteria. Front. Cell. Infect. Microbiol. 7. doi: 10.3389/fcimb.2017.00165 PMC5418226 28529926

[B29] Schwarz E. M. Parvizi J. Gehrke T. Aiyer A. Battenberg A. Brown S. A.. (2019). 2018 international consensus meeting on musculoskeletal infection: research priorities from the general assembly questions. J. Orthop Res. 37, 997–1006. doi: 10.1002/jor.24293 30977537

[B30] Sheedy F. J. Grebe A. Rayner K. J. Kalantari P. Ramkhelawon B. Carpenter S. B.. (2013). CD36 coordinates NLRP3 inflammasome activation by facilitating intracellular nucleation of soluble ligands into particulate ligands in sterile inflammation. Nat. Immunol. 14, 812–820. doi: 10.1038/ni.2639 23812099 PMC3720827

[B31] Siddiqui R. Suzu S. Ueno M. Nasser H. Koba R. Bhuyan F.. (2018). Apolipoprotein e is an HIV-1-inducible inhibitor of viral production and infectivity in macrophages. PloS Pathog. 14, e1007372. doi: 10.1371/journal.ppat.1007372 30496280 PMC6289579

[B32] Smeltzer M. S. Thomas J. R. Hickraon S. G. Skinner R. A. Nelson C. L. Griffith D.. (1997). Characterization of a rabbit model of staphylococcal osteomyelitis. J. Orthopaedic Res. 15, 414–421. doi: 10.1002/jor.1100150314 9246088

[B33] Soh K. Y. Loh J. M. S. Proft T. (2020). Cell wall-anchored 5’-nucleotidases in gram-positive cocci. Mol. Microbiol. 113, 691–698. doi: 10.1111/mmi.14442 31872460

[B34] Takeda K. Kaisho T. Akira S. (2003). Toll-like receptors. Annu. Rev. Immunol. 21, 335–376. doi: 10.1146/annurev.immunol.21.120601.141126 12524386

[B35] Tavazoie M. F. Pollack I. Tanqueco R. Ostendorf B. N. Reis B. S. Gonsalves F. C.. (2018). LXR/ApoE activation restricts innate immune suppression in cancer. Cell. 172, 825–840.e18. doi: 10.1016/j.cell.2017.12.026 29336888 PMC5846344

[B36] Tenger C. Zhou X. (2003). Apolipoprotein e modulates immune activation by acting on the antigen-presenting cell. Immunology. 109, 392–397. doi: 10.1046/j.1365-2567.2003.01665.x 12807485 PMC1782975

[B37] Wang Q. Huang J. Li. S. Zhang Y. Sun. R. Ren. J.. (2022). Fermentation supernatant of staphylococcus aureus drives catabolism in chondrocytes via NF-κB signaling mediated increase of cholesterol metabolism. Exp. Cell Res. 410(1):112952. doi: 10.1016/j.yexcr.2021.112952 34848206

[B38] West A. P. Brodsky I. E. Rahner C. Woo D. K. Erdjument-Bromage H. Tempst P.. (2011). TLR signalling augments macrophage bactericidal activity through mitochondrial ROS. Nature. 472, 476–480. doi: 10.1038/nature09973 21525932 PMC3460538

[B39] Wynn T. A. Chawla A. Pollard J. W. (2013). Macrophage biology in development, homeostasis and disease. Nature. 496, 445–455. doi: 10.1038/nature12034 23619691 PMC3725458

[B40] Xiao J. Li W. Zheng X. Qi L. Wang H. Zhang C.. (2020). Targeting 7-dehydrocholesterol reductase integrates cholesterol metabolism and IRF3 activation to eliminate infection. Immunity. 52, 109–122.e6. doi: 10.1016/j.immuni.2019.11.015 31882361

[B41] Zhang S. H. Reddick R. L. Piedrahita J. A. Maeda N. (1992). Spontaneous hypercholesterolemia and arterial lesions in mice lacking apolipoprotein e. Science. 258, 468–471. doi: 10.1126/science.1411543 1411543

[B42] Zhao G. Mo Z.-C. Tang S.-L. Ouyang X.-P. He P. Lv Y.. (2014). Chlamydia pneumoniae negatively regulates ABCA1 expression via TLR2-nuclear factor-kappa b and miR-33 pathways in THP-1 macrophage-derived foam cells. Atherosclerosis. 235, 519–525. doi: 10.1016/j.atherosclerosis.2014.05.943 24953492

[B43] Zhu Y. Nwabuisi-Heath E. Dumanis S. B. Tai L. M. Yu C. Rebeck G. W.. (2012). APOE genotype alters glial activation and loss of synaptic markers in mice. Glia. 60, 559–569. doi: 10.1002/glia.22289 22228589 PMC3276698

