# Supplementary material for: Apolipoprotein E deficiency potentiates macrophage against Staphylococcus aureus in mice with osteomyelitis via regulating cholesterol metabolism
Source: Front Cell Infect Microbiol. 2023 Jul 17;13:1187543. doi: 10.3389/fcimb.2023.1187543 (PMC10387542; doi:10.3389/fcimb.2023.1187543)
Supplement: Supplementary file 1 [file DataSheet_1.docx]

# Supplementary Figure 1

**
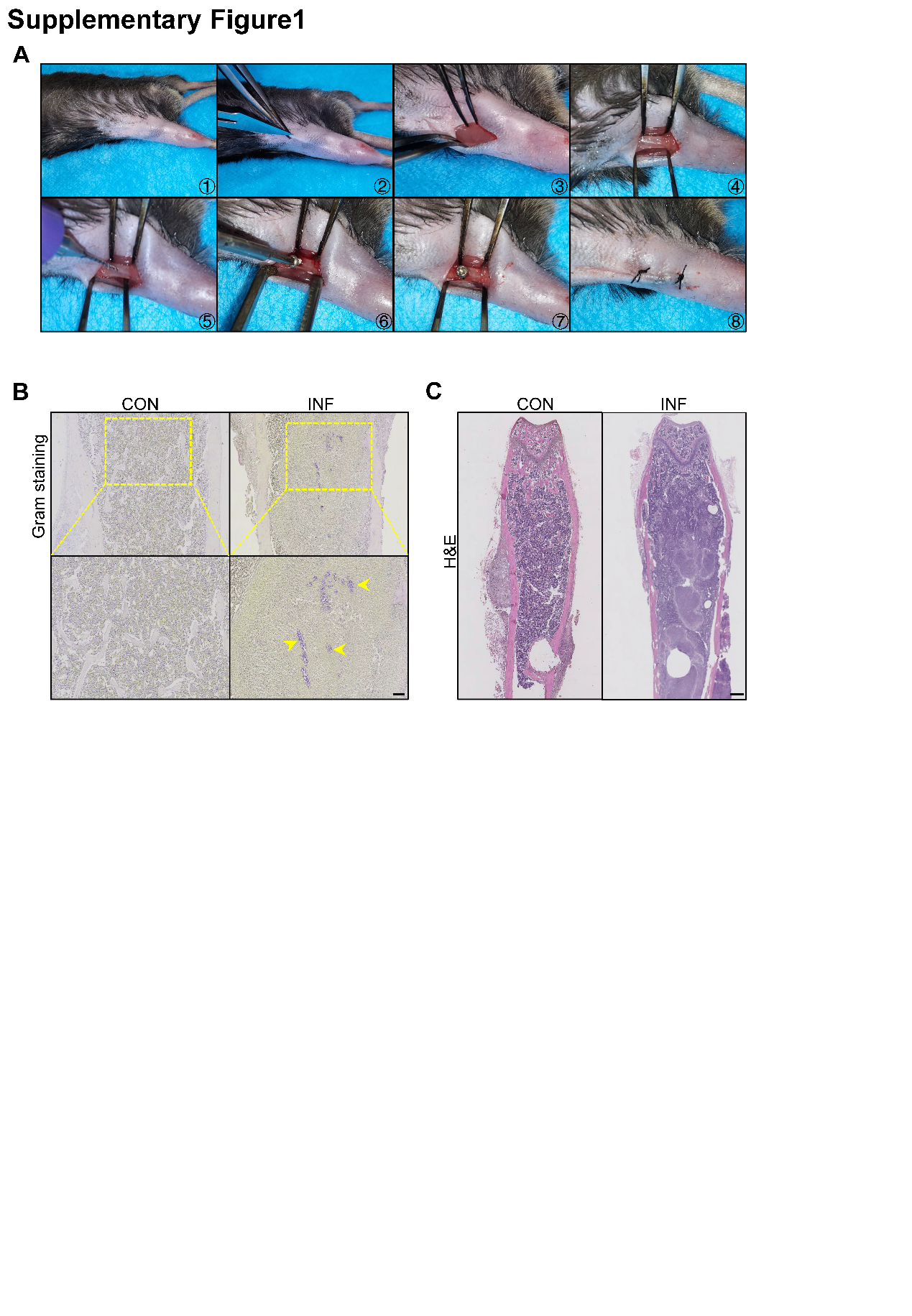
**

**Modified implant-associated osteomyelitis mouse model**

(A)The establishment of Modified implant-associated osteomyelitis mouse model.(B) Representative images of gram staining of femurs in mice with or without *S. aureus* osteomyelitis by days 7 postinfection. Yellow arrows indicate *S. aureus*. Scale bar was 100um*.* (C) Representative images of H&E staining of femurs in mice with or without *S. aureus* osteomyelitis by days 7 postinfection. Scale bar was 500um.

**Supplementary Figure 2**


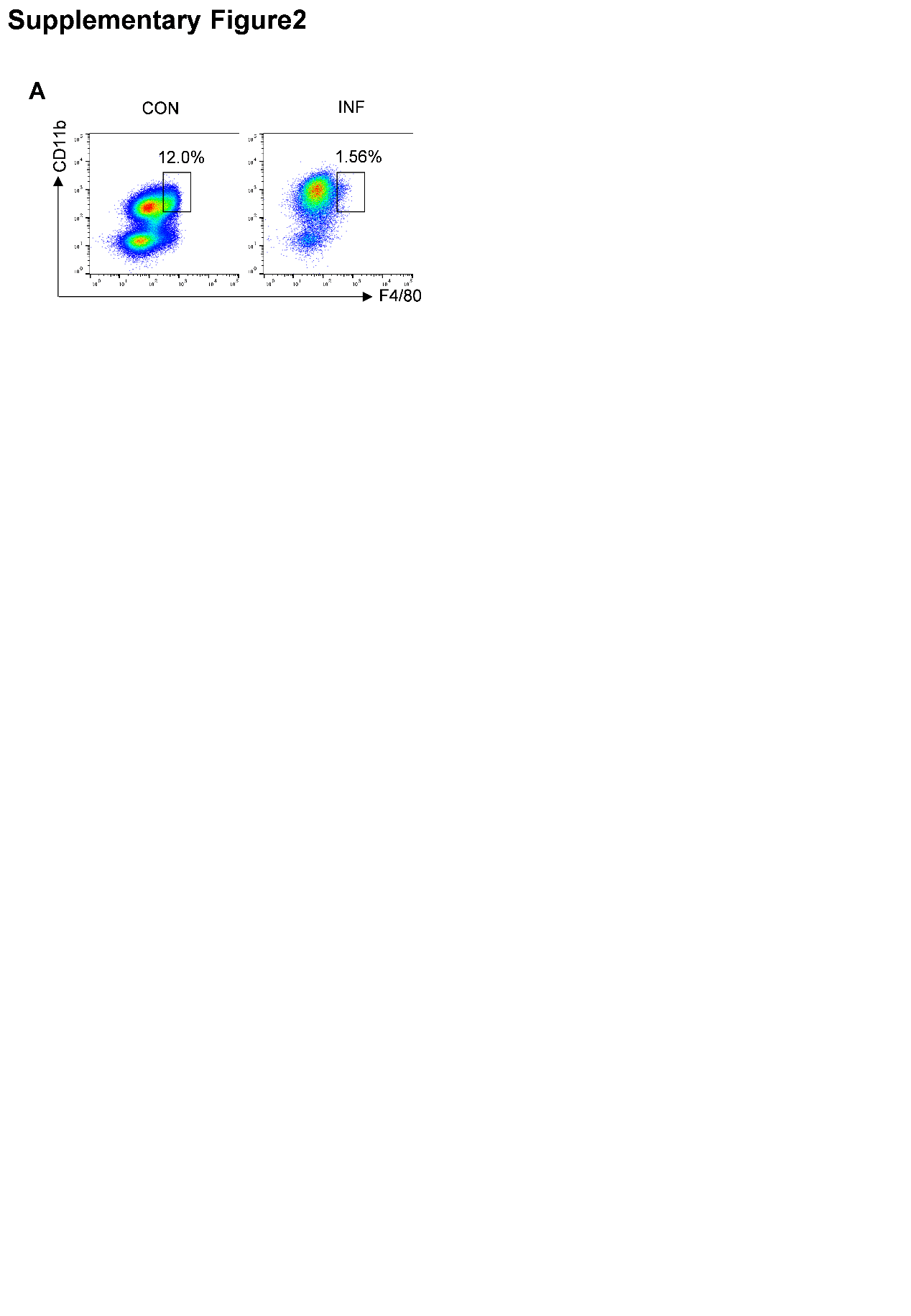


***S. aureus* infection decreased the proportion of bone marrow macrophages**

(A)Representative images of flow cytometry for proportion of CD11b^+^F4/80^+^ macrophages from bone marrow of *S. aureus* osteomyelitis mice and controls, respectively, by day 7 post-surgery.

**Supplementary Figure 3**

**
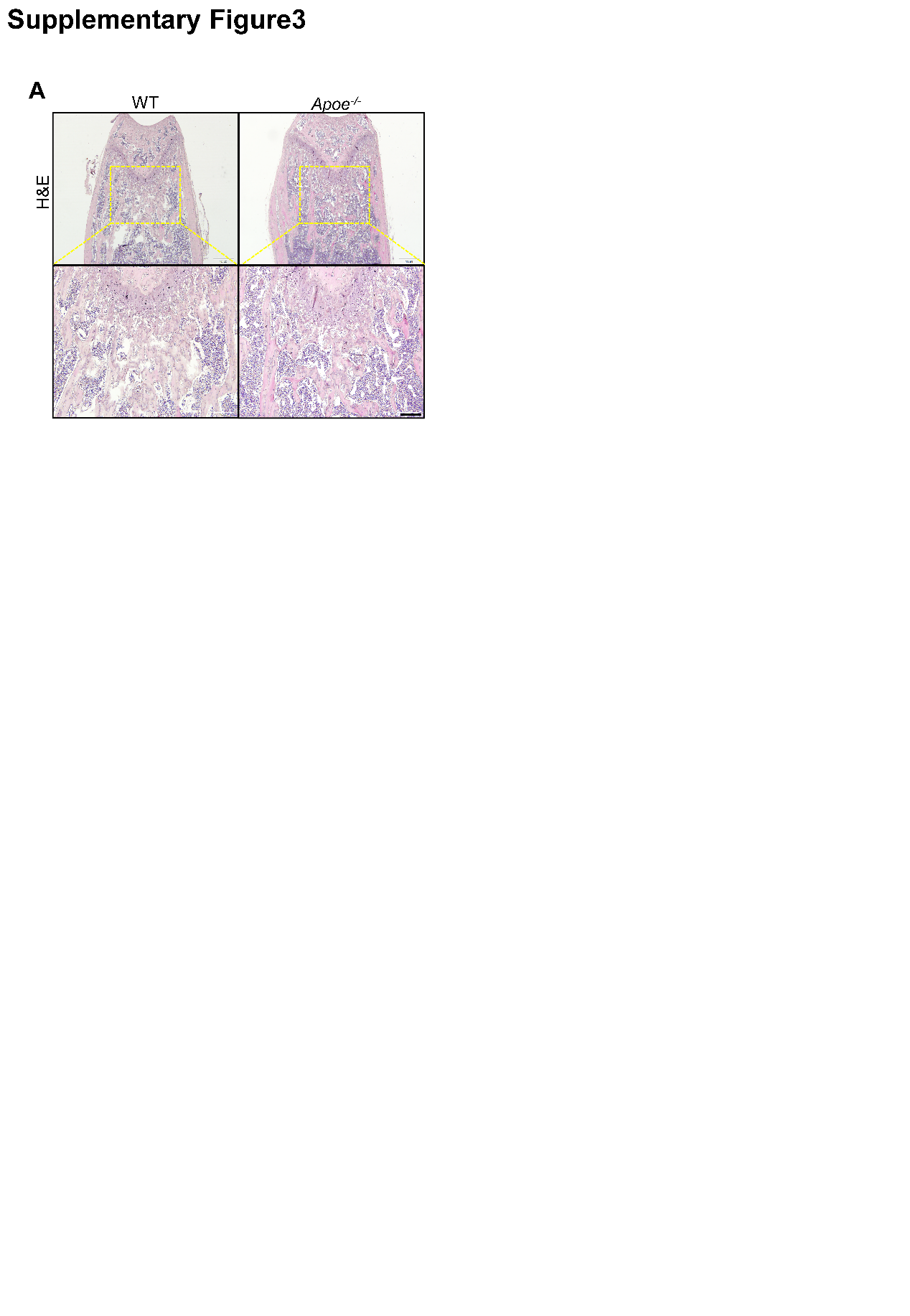
**

**The effect of APOE deficiency on bone mass in 8-week-old male mice** (A)Representative H&E staining of femurs from 8-week-old male WT or *Apoe^-/-^* mice. Scale bar was 100um.

# Supplementary Table 1

Collection of cholesterol metabolism gene set

| **Pathway** |
| --- |
| BIOCARTA_FXR_PATHWAY |
| CHEN_LIVER_METABOLISM_QTL_CIS |
| GOBP_CELLULAR_RESPONSE_TO_CHOLESTEROL |
| GOBP_CORTISOL_METABOLIC_PROCESS |
| GOBP_HIGH_DENSITY_LIPOPROTEIN_PARTICLE_REMODELING |
| GOBP_LOW_DENSITY_LIPOPROTEIN_PARTICLE_REMODELING |
| GOBP_NEGATIVE_REGULATION_OF_CHOLESTEROL_EFFLUX |
| GOBP_NEGATIVE_REGULATION_OF_CHOLESTEROL_METABOLIC_PROCESS |
| GOBP_NEGATIVE_REGULATION_OF_CHOLESTEROL_STORAGE |
| GOBP_POSITIVE_REGULATION_OF_CHOLESTEROL_EFFLUX |
| GOBP_POSITIVE_REGULATION_OF_CHOLESTEROL_ESTERIFICATION |
| GOBP_POSITIVE_REGULATION_OF_CHOLESTEROL_METABOLIC_PROCESS |
| GOBP_POSITIVE_REGULATION_OF_CHOLESTEROL_STORAGE |
| GOBP_RECEPTOR_MEDIATED_ENDOCYTOSIS_INVOLVED_IN_CHOLESTEROL_TRANSPORT |
| GOBP_REGULATION_OF_CHOLESTEROL_EFFLUX |
| GOBP_REGULATION_OF_CHOLESTEROL_ESTERIFICATION |
| GOBP_REGULATION_OF_CHOLESTEROL_METABOLIC_PROCESS |
| GOBP_REGULATION_OF_RECEPTOR_MEDIATED_ENDOCYTOSIS_INVOLVED_IN_CHOLESTEROL_TRANSPORT |
| GOBP_RESPONSE_TO_CHOLESTEROL |
| GOBP_RESPONSE_TO_MINERALOCORTICOID |
| GOBP_TRIGLYCERIDE_RICH_LIPOPROTEIN_PARTICLE_REMODELING |
| GOCC_CHYLOMICRON |
| GOCC_HIGH_DENSITY_LIPOPROTEIN_PARTICLE |
| GOCC_LOW_DENSITY_LIPOPROTEIN_PARTICLE |
| GOCC_SPHERICAL_HIGH_DENSITY_LIPOPROTEIN_PARTICLE |
| GOCC_TRIGLYCERIDE_RICH_PLASMA_LIPOPROTEIN_PARTICLE |
| GOERING_BLOOD_HDL_CHOLESTEROL_QTL_CIS |
| GOERING_BLOOD_HDL_CHOLESTEROL_QTL_TRANS |
| GOMF_CHOLESTEROL_BINDING |
| GOMF_LOW_DENSITY_LIPOPROTEIN_PARTICLE_BINDING |
| GOMF_PHOSPHATIDYLCHOLINE_STEROL_O_ACYLTRANSFERASE_ACTIVATOR_ACTIVITY |
| HALLMARK_CHOLESTEROL_HOMEOSTASIS |
| HORTON_SREBF_TARGETS |
| HP_ABNORMAL_CIRCULATING_CHOLESTEROL_CONCENTRATION |
| HP_ABNORMAL_HDL_CHOLESTEROL_CONCENTRATION |
| HP_ABNORMAL_LDL_CHOLESTEROL_CONCENTRATION |
| HP_ABNORMALITY_OF_LIPOPROTEIN_CHOLESTEROL_CONCENTRATION |
| HP_DECREASED_LDL_CHOLESTEROL_CONCENTRATION |
| HP_HYPERCHOLESTEROLEMIA |
| HP_HYPERLIPOPROTEINEMIA |
| HP_HYPOCHOLESTEROLEMIA |
| HP_HYPOLIPOPROTEINEMIA |
| HP_INCREASED_LDL_CHOLESTEROL_CONCENTRATION |
| HP_INCREASED_VLDL_CHOLESTEROL_CONCENTRATION |
| KEGG_PRIMARY_BILE_ACID_BIOSYNTHESIS |
| KEGG_STEROID_HORMONE_BIOSYNTHESIS |
| LIPID_RAFT |
| REACTOME_CHOLESTEROL_BIOSYNTHESIS |
| REACTOME_NR1H2_NR1H3_REGULATE_GENE_EXPRESSION_TO_LIMIT_CHOLESTEROL_UPTAKE |
| REACTOME_NR1H3_NR1H2_REGULATE_GENE_EXPRESSION_LINKED_TO_CHOLESTEROL_TRANSPORT_AND_EFFLUX |
| REACTOME_REGULATION_OF_CHOLESTEROL_BIOSYNTHESIS_BY_SREBP_SREBF |
| WP_CHOLESTEROL_BIOSYNTHESIS_PATHWAY |
| WP_CHOLESTEROL_BIOSYNTHESIS_WITH_SKELETAL_DYSPLASIAS |
| WP_CHOLESTEROL_METABOLISM_WITH_BLOCH_AND_KANDUTSCHRUSSELL_PATHWAYS |
| WP_MEVALONATE_ARM_OF_CHOLESTEROL_BIOSYNTHESIS_PATHWAY |
| WP_OXYSTEROLS_DERIVED_FROM_CHOLESTEROL |
| WP_SREBF_AND_MIR33_IN_CHOLESTEROL_AND_LIPID_HOMEOSTASIS |
| WP_STATIN_INHIBITION_OF_CHOLESTEROL_PRODUCTION |
| GOBP_CORTISOL_BIOSYNTHETIC_PROCESS |
| GOBP_CELLULAR_RESPONSE_TO_MINERALOCORTICOID_STIMULUS |
| WUNDER_INFLAMMATORY_RESPONSE_AND_CHOLESTEROL_DN |
| WUNDER_INFLAMMATORY_RESPONSE_AND_CHOLESTEROL_UP |
| GOBP_POSITIVE_REGULATION_OF_CHOLESTEROL_BIOSYNTHETIC_PROCESS |
| GOBP_REGULATION_OF_CHOLESTEROL_BIOSYNTHETIC_PROCESS |
| GOBP_VITAMIN_D3_METABOLIC_PROCESS |
| GOBP_POLYOL_BIOSYNTHETIC_PROCESS |
| GOBP_STEROL_IMPORT |
| GOBP_CELLULAR_RESPONSE_TO_STEROL_DEPLETION |
| GOBP_NEGATIVE_REGULATION_OF_STEROL_TRANSPORT |
| GOMF_OXIDOREDUCTASE_ACTIVITY_ACTING_ON_THE_ALDEHYDE_OR_OXO_GROUP_OF_DONORS_NAD_OR_NADP_AS_ACCEPTOR |
| GOBP_VESICLE_MEDIATED_CHOLESTEROL_TRANSPORT |
| GOMF_OXIDOREDUCTASE_ACTIVITY_ACTING_ON_PAIRED_DONORS_WITH_INCORPORATION_OR_REDUCTION_OF_MOLECULAR_OXYGEN_NAD_P_H_AS_ONE_DONOR_AND_INCORPORATION_OF_ONE_ATOM_OF_OXYGEN |
| GOBP_CHOLESTEROL_STORAGE |
| GOBP_VITAMIN_BIOSYNTHETIC_PROCESS |
| GOMF_OXIDOREDUCTASE_ACTIVITY_ACTING_ON_THE_ALDEHYDE_OR_OXO_GROUP_OF_DONORS |
| GOMF_OXIDOREDUCTASE_ACTIVITY_ACTING_ON_CH_OH_GROUP_OF_DONORS |
| GOBP_POSITIVE_REGULATION_OF_STEROL_TRANSPORT |
| GOMF_STEROL_TRANSFER_ACTIVITY |
| GOBP_INTRACELLULAR_STEROL_TRANSPORT |
| GOBP_CHOLESTEROL_CATABOLIC_PROCESS |
| GOBP_FAT_SOLUBLE_VITAMIN_CATABOLIC_PROCESS |
| GOMF_STEROID_HYDROXYLASE_ACTIVITY |
| GOBP_INTRACELLULAR_LIPID_TRANSPORT |
| GOMF_OXYSTEROL_BINDING |
| GOBP_CHOLESTEROL_EFFLUX |
| GOBP_CELLULAR_RESPONSE_TO_STEROL |
| GOBP_STEROID_CATABOLIC_PROCESS |
| GOBP_ORGANIC_HYDROXY_COMPOUND_CATABOLIC_PROCESS |
| GOBP_REVERSE_CHOLESTEROL_TRANSPORT |
| GOBP_FAT_SOLUBLE_VITAMIN_METABOLIC_PROCESS |
| GOBP_FAT_SOLUBLE_VITAMIN_BIOSYNTHETIC_PROCESS |
| GOBP_STEROL_HOMEOSTASIS |
| GOBP_RESPONSE_TO_STEROL |

**Supplementary Table 2**

PCR primers

| Beta-Actin,forward | GGCTGTATTCCCCTCCATCG |
| --- | --- |
| Beta-Actin,reverse | CCAGTTGGTAACAATGCCATGT |
| APOE,forward | AGAACTGACGGCACTGATGG |
| APOE, reverse | AGAGACTCAGAATGTGCTCGG |
| ABCA1,forward | ATTCAGCTTGGTGATGCGGA |
| ABCA1, reverse | TGGGTCGGGAGATGAGATGT |
| SQLE,forward | CGACAGGATAGTTGGGGAGC |
| SQLE, reverse | TCCTTGTATTGCACGCCGAT |
| LSS,forward | TCGTGGGGGACCCTATAAAAC |
| LSS, reverse | CGTCCTCCGCTTGATAATAAGTC |
| CYP51,forward | GACAGGAGGCAACTTGCTTTC |
| CYP51, reverse | GTGGACTTTTCGCTCCAGC |
| DHCR24,forward | CTCTGGGTGCGAGTGAAGG |
| DHCR24, reverse | TTCCCGGACCTGTTTCTGGAT |

# Supplementary methods

**Cholesterol measurement of bone marrow macrophages by LC-MS/MS based metabolomics approach**

The cell sample stored at -80 °C refrigerator was thawed on ice. A 500 μL solution (Methanol: Water = 4:1, V/V) containing internal standard was added into the cell sample and vortexed for 3 min. The sample was placed in liquid nitrogen for 5 min and on the dry ice for 5 min, and then thawed on ice and vortexed for 2 min. This freeze-thaw circle was repeated three times in total. The sample was centrifuged at 12000 rpm for 10 min (4°C). A 300 μL of supernatant was collected and placed in -20°C for 30 min. The sample was then centrifuged at 12000 rpm for 3 min (4 °C). A 200 μL aliquots of supernatant were transferred for LC-MS analysis.

The sample extracts were analyzed using an LC-ESI-MS/MS system (UPLC, ExionLC AD，<https://sciex.com.cn/>; MS, QTRAP® System, <https://sciex.com/>). The analytical conditions were as follows, UPLC: column, Waters ACQUITY UPLC HSS T3 C18 (1.8μm, 2.1 mm*100 mm); column temperature, 40°C; flow rate, 0.4 mL/min; injection volume, 2μL or 5μL; solvent system, water (0.1% formic acid): acetonitrile (0.1% formic acid); gradient program, 95:5 V/V at 0 min, 10:90 V/V at 10.0 min, 10:90 V/V at 11.0 min, 95:5 V/V at 11.1 min, 95:5 V/V at 14.0 min.

The Triple TOF mass spectrometer was used for its ability to acquire MS/MS spectra on an information-dependent basis (IDA) during an LC/MS experiment. In this mode, the acquisition software (TripleTOF 6600, AB SCIEX) continuously evaluates the full scan survey MS data as it collects and triggers the acquisition of MS/MS spectra depending on preselected criteria. In each cycle, 12 precursor ions whose intensity greater than 100 were chosen for fragmentation at collision energy (CE) of 30 V (12 MS/MS events with product ion accumulation time of 50 msec each). ESI source conditions were set as following: Ion source gas 1 as 50 Psi, Ion source gas 2 as 50 Psi, Curtain gas as 25 Psi, source temperature 500°C, Ion Spray Voltage Floating (ISVF) 5500 V or -4500 V in positive or negative modes, respectively.

LIT and triple quadrupole (QQQ) scans were acquired on a triple quadrupole-linear ion trap mass spectrometer (QTRAP), QTRAP® LC-MS/MS System, equipped with an ESI Turbo Ion-Spray interface, operating in positive and negative ion mode and controlled by Analyst 1.6.3 software (Sciex). The ESI source operation parameters were as follows: source temperature 500°C; ion spray voltage (IS) 5500 V (positive), -4500 V (negative); ion source gas I (GSI), gas II (GSII), curtain gas (CUR) were set at 50, 50, and 25.0 psi, respectively; the collision gas (CAD) was high. Instrument tuning and mass calibration were performed with 10 and 100 μmol/L polypropylene glycol solutions in QQQ and LIT modes, respectively. A specific set of MRM transitions were monitored for each period according to the metabolites eluted within this period.

**Cholesterol measurement of bone marrow supernatant by LC-MS/MS based metabolomics approach**

The supernatant sample was taken out from the -80 °C refrigerator, thawed on ice and vortexed for 10 s. Mix 200 μL of the sample and 1mL of the extraction solvent (MTBE: MeOH =3:1, v/v) containing internal standard mixture. After whirling the mixture for 15 min, 100 μL of water was added. Vortex for 1 min and centrifuge at 12,000 rpm for 10 min. 500 μL of the upper organic layer was collected and evaporated using a vacuum concentrator. The dry extract was reconstituted using 200 μL mobile phase B prior to LC-MS/MS analysis.

The sample extracts were analyzed using an LC-ESI-MS/MS system (UPLC, ExionLC AD, <https://sciex.com.cn/>; MS, QTRAP® System, <https://sciex.com/>). The analytical conditions were as follows, UPLC: column, Thermo Accucore™ C30 (2.6 μm, 2.1 mm*100 mm i.d.); solvent system, A: acetonitrile/water (60/40,V/V, 0.1% formic acid, 10 mmol/L ammonium formate), B: acetonitrile/isopropanol (10/90 V/V, 0.1% formic acid, 10 mmol/L ammonium formate); gradient program, A/B (80:20, V/V) at 0 min, 70:30 V/V at 2.0 min, 40:60 V/V at 4 min, 15:85 V/V at 9 min, 10:90 V/V at 14 min, 5:95 V/V at 15.5 min, 5:95 V/V at 17.3 min, 80:20 V/V at 17.3 min, 80:20 V/V at 20 min; flow rate, 0.35 ml/min; temperature, 45 °C; Injection volume: 2μl. The effluent was alternatively connected to an ESI-triple quadrupole-linear ion trap (QTRAP)-MS.

LIT and triple quadrupole (QQQ) scans were acquired on a triple quadrupole-linear ion trap mass spectrometer (QTRAP), QTRAP® LC-MS/MS System, equipped with an ESI Turbo Ion-Spray interface, operating in positive and negative ion mode and controlled by Analyst 1.6.3 software (Sciex). The ESI source operation parameters were as follows: ion source, turbo spray; source temperature 500 °C; ion spray voltage (IS) 5500 V (Positive), -4500 V(Neagtive); ion source gas 1 (GS1), gas 2 (GS2), curtain gas (CUR) were set at 45, 55, and 35 psi, respectively; the collision gas (CAD) was medium. Instrument tuning and mass calibration were performed with 10 and 100 μmol/L polypropylene glycol solutions in QQQ and LIT modes, respectively. QQQ scans were acquired as MRM experiments with collision gas (nitrogen) set to 5 psi. DP and CE for individual MRM transitions was done with further DP and CE optimization. A specific set of MRM transitions were monitored for each period according to the metabolites eluted within this period.

**RNA preparation and transcriptome sequencing**

Total RNA of the bone marrow was extracted with TRIzol reagent according to the manufacturer’s instructions (Invitrogen, USA).RNA degradation and contamination was monitored on 1% agarose gels.RNA purity was checked using the NanoPhotometer® spectrophotometer (IMPLEN, CA, USA) . RNA concentration was measured using Qubit® RNA Assay Kit in Qubit®2.0 Flurometer (Life Technologies, CA, USA).RNA integrity was assessed using the RNA Nano 6000 Assay Kit of the Bioanalyzer 2100 system(Agilent Technologies, CA, USA)

For transcriptome sequencing, a total amount of 1 µg RNA per sample was used as input material for the RNA sample preparations. Sequencing libraries were generated using NEBNext® UltraTM RNA Library Prep Kit for Illumina® (NEB, USA) following manufacturer’s recommendations and index codes were added to attribute sequences to each sample. Briefly, mRNA was purified from total RNA using poly-T oligo attached magnetic beads. Fragmentation was carried out using divalent cations under elevated temperature in NEBNext First Strand Synthesis Reaction Buffer(5X). First strand cDNA was synthesized using random hexamer primer and M-MuLV Reverse Transcriptase (RNase H-). Second strand cDNA synthesis was subsequently performed using DNA Polymerase I and RNase H. Remaining overhangs were converted into blunt ends via exonuclease/polymerase activities. After adenylation of 3’ ends of DNA fragments, NEBNext Adaptor with hairpin loop structure were ligated to prepare for hybridization. In order to select cDNA fragments of preferentially 250~300 bp in length, the library fragments were purified with AMPure XP system (Beckman Coulter, Beverly, USA). Then 3 µl USER Enzyme (NEB, USA) was used with size-selected, adaptor-ligated cDNA at 37°C for 15 min followed by 5 min at 95 °C before PCR. Then PCR was performed with Phusion High-Fidelity DNA polymerase, Universal PCR primers and Index (X) Primer. PCR products were purified (AMPure XP system) and library quality was assessed on the Agilent Bioanalyzer 2100 system. The clustering of the index-coded samples was performed on a cBot Cluster Generation System using TruSeq PE Cluster Kit v3-cBot-HS (Illumia) according to the manufacturer’s instructions. After cluster generation, the library preparations were sequenced on an Illumina Hiseq platform and 125 bp/150 bp paired-end reads were generated.
